# The Suicidal Animal: Science and the Nature of Self-Destruction*

**DOI:** 10.1093/pastj/gtu015

**Published:** 2014-07-24

**Authors:** Edmund Ramsden, Duncan Wilson

**Affiliations:** Queen Mary, University of London; University of Manchester

## I

### INTRODUCTION

In 1897, the French sociologist Emile Durkheim published *Suicide*, his renowned statistical study that sought to categorize the varying forms of self-destruction as egoistic, fatalistic, anomic or altruistic. Durkheim built upon the work of the English jurist William Wynn Westcott and the Italian psychiatrist Enrico Morselli, who both perceived suicide as a social phenomenon. Although he did not dispute that the vast majority of suicides (barring neurasthenics) intentionally took their own lives, Durkheim argued their actions should be properly regarded as ‘confirmation of a resolve previously formed for reasons unknown to consciousness’.^1^ The stability of suicide rates, he concluded, demonstrated the ‘existence of collective tendencies exterior to the individual’.^2^ For the historian Olive Anderson, *Suicide* is thus emblematic of the *fin de siècle* view that suicide was a social problem — marking a crucial break from the Romantic belief that it was a supremely individualistic act.^3^ Stephen Turner, meanwhile, portrays Durkheim as the ‘prodigal child’ of nineteenth-century positivism, in that he believed statistics could illuminate the ‘underlying causal order that determined human actions’.^4^ And Daryl Lee has shown how Durkheim believed that this approach would elucidate the ‘deep disturbance from which civilized societies are suffering’ and, in so doing, advance sociology as the pre-eminent means of diagnosing the various ills that plagued modernity.^5^

But for all the justifiable attention Durkheim’s work has received, these historians have overlooked one striking aspect of *Suicide*. At the book’s outset, Durkheim defined suicide as ‘all cases of death resulting directly or indirectly from a positive or negative act of the victim himself, which he knows will produce this result’.^6^ He then outlined precisely why this definition ‘excludes from our study everything related to the suicide of animals’.^7^ Contemporary knowledge of the animal mind, Durkheim argued, ‘does not really attribute to them an understanding anticipatory of their death nor, especially, of the means to accomplish it’. Instances where animals seemingly killed themselves, he continued,

may be quite differently explained. If the irritated scorpion pierces itself with its sting (which is not at all certain) it is probably from an automatic, unreflecting reaction. The motive energy caused by his irritation is discharged by chance and at random; the creature happens to become its victim, though it cannot be said to have had a preconception of the result of its action.^8^

Similarly, Durkheim stated that if dogs starved to death after the loss of their masters, ‘it is because the sadness into which they are thrown has automatically caused lack of hunger; death has resulted, but without having been foreseen’. Since neither the scorpion nor the dog used self-injury or fasting ‘as a means to a known effect’, he concluded, ‘the special characteristics of suicide as defined by us are lacking’.^9^

Durkheim’s refutation of the suicidal animal may seem to us straightforward. Yet why did he even engage with the issue, if only to dismiss it? Answering this question draws our attention to a hitherto neglected aspect of the history of suicide. While it may seem obvious to many that suicide is a uniquely human act, this belief has a history; and it is a history built upon reflections on the natural world. In order to present suicide as uniquely human, Durkheim had to discuss the seemingly deficient animal mind. In doing so, he engaged in a rhetorical ploy common to many treatises on suicide, which elevate humans above animals on account of their ability consciously to reflect upon life and death, and then choose self-destruction.

For Albert Camus, like Durkheim, anguishing over whether or not to end one’s life was precisely what set humanity apart from the natural world. ‘Were I a cat among cats’, Camus stated in 1942, ‘this problem would not arise, for I would belong to this world. I should *be* in this world to which I am now opposed by my whole consciousness’.^10^ This, then, is a world view dependent on that which it seeks to exclude, and we are reminded of Jacques Derrida’s notion of the *supplement*: where something apparently ephemeral, on the fringes of a subject, is actually fundamental to it.^11^ And it bears out Erica Fudge’s claim that the qualities used to define the human — such as speech, rational thought or suicide — can only be rendered meaningful through reference to animals. ‘To explain the human’, Fudge argues, is ‘to explain the animal; or perhaps that should be reversed: to explain the animal is to explain the human’.^12^

Although animals have recently moved to a more central position in historical work, historians have tended to restrict their attention to the ways in which reflections on animals have underpinned an anthropocentric world view. Esther Cohen, Erica Fudge, Keith Thomas and others have shown how medieval, early modern and Enlightenment authorities distinguished humans from the natural world by representing animals as insensate and irrational.^13^ While this would appear to be the case with *Suicide*, it is by no means the whole story. By arguing that animals did not intentionally kill themselves, Durkheim was not simply establishing the boundaries of his inquiry. He was also seeking to refute contemporary writers who problematized the anthropocentric world view by arguing that animals *did commit suicide*. Charting these arguments in favour of the existence of animal suicide, we believe, provides a window onto a most enduring and potent challenge to human exceptionalism.

In this article, we first outline how support for belief in animal suicide reflected, and linked, social and scientific concerns during the late nineteenth century. Advocates included anti-cruelty campaigners and medical reformers, who sought to inculcate sympathy for both man and beast, and supporters of evolution by common descent, who endorsed continuity between the animal and human minds. We show how these writers, who included psychiatrists and psychologists, claimed that animals and humans both possessed the ability to consciously plan and execute their own deaths. These accounts, we argue, reflected both the Romantic view that suicide was a rational and individualistic act and, slightly later, the medical belief that it stemmed from ‘temporary insanity’. We then show how there was a shift in focus from the late nineteenth century onwards. Here, attention turned to accounts of mass animal suicide, betraying a growing belief that the *fin de siècle* period was marked by a ‘universal wish not to live’, in which human self-destruction was a trend, process or ‘disease of civilization’.^14^ Within this shift, the archetype of animal suicide was transposed from the romanticized or insane individual to the anomic population. As a consequence, the endangered scorpion and grieving dog discussed in *Suicide* were usurped by the twentieth century’s emblematic animal suicide: the lemming.

We will examine how a shift from suicide viewed as an individual and intentional act, to a complex of self-destructive behaviours determined by various social and biological forces, was influenced by the growing importance of the animal laboratory to the study of population dynamics and psychopathology. While attempts to induce suicide in laboratory animals in the nineteenth century were used to dismiss anthropomorphic anecdotes about animals dying in defiance, anger or grief, laboratory studies promoted the understanding of behaviour in terms of mechanical and physiological responses to stimuli. By the mid twentieth century, a zoomorphic perspective had not only become paradigmatic in fields that investigated the animal mind and behaviour, such as psychology, but also influenced work in psychiatry and population studies. Scholars in these fields now interpreted self-destructive behaviour, in humans and animals, in terms of innate and unconscious responses to social and ecological pressures.

In our final section, we explore how the ecological study of lemming behaviour dovetailed with an emergent field of experimental psychiatry, which sought to provide the study of psychopathology with a rigorous scientific basis. Together, they promoted stress models of suicide among humans and animals. It was through the concept of *stress*, we argue, that long-standing divisions were overcome and tensions resolved: between the individual and the collective, intention and automation, and instincts of self-destruction and self-preservation.

## II

### FROM ANECDOTE TO EXPERIMENT: THE SCIENCE OF ANIMAL SUICIDE IN THE 1870S AND 1880S

Discussion of animal self-destruction during the early nineteenth century was structured by, and perpetuated, the Romantic view that suicide was a rational and even noble escape from intolerable circumstances. Popular accounts generally concerned animals that intentionally ended their lives to escape hopeless danger or human mistreatment. Prominent among these was the scorpion, which, when ringed with fire and faced with no means of escape, was said to kill itself by thrusting its sting into its own back. Although scorpion suicide had long featured in Iberian folklore, it was popularized by George Byron’s 1813 poem *The Giaour*. Like other Romantics, Byron portrayed suicide as a natural and heroic act, and regularly asserted his kinship with animals.^15^ In *The Giaour*, he framed scorpion suicide as a perfect analogy for the inner torment of the human condition. ‘The mind that broods o’er guilty woes’, he wrote, ‘Is like the scorpion girt by fire’. With clear human inference, he outlined how endangered scorpions intentionally chose self-destruction:

In circle narrowing as it glows

The flames around their captive close,

Till inly search’d by thousand throes,

And maddening in her ire,

One sad and sole relief she knows,

The sting she nourished for her foes,

Whose venom never yet was vain

Gives but one pang and cures all pain,

And darts into her desperate brain. —

So do the dark in soul expire

Or live like the scorpion girt by fire.^16^

Byron’s ‘scorpion girt by fire’ would later become the first experimental model for animal suicide, and was eventually used to refute Romantic and anthropomorphic perspectives on self-destruction. But the scorpion was by no means the only suicidal animal to feature in popular debates during the nineteenth century. As Keith Thomas and Kathleen Kete have both detailed, increasing pet ownership and domestication of working animals fostered many popular accounts of animal intelligence, or ‘sagacity’.^17^ Newspaper accounts of animal suicide formed a small but striking group of these reports, generally appearing in the provincial press or in the country section of metropolitan newspapers. In 1845, for example, *The Satirist* reported that a ‘fine’ Newfoundland dog had recently committed suicide in Holmfirth, Yorkshire, by drowning itself in a river. The report outlined how each time the dog was ‘repeatedly dragged out, it was no sooner released than it again rushed in, and at last determinedly held its head under water until life was extinct’.^18^ When the naturalist Edward Jesse detailed this incident in an 1858 edition of his *Anecdotes of Dogs*, he notably stressed that the suicide, with its planning and ‘repeated efforts’, offered definitive ‘proof of the general instinct and sagacity of the canine race’.^19^

Neither *The Satirist* nor Jesse provided any mention of motive for this dog’s apparent suicide. But this was not the case with the growing number of reports that circulated during the 1870s and 1880s. These brought together a number of concerns in late nineteenth-century Britain. They reflected, on the one hand, the efforts of anti-cruelty campaigners who sought to improve human behaviour towards animals by showing that humans and animals possessed similar emotional and intellectual capacities. In its regular journal *The Animal World*, the Royal Society for the Protection of Animals (RSPCA) echoed *The Giaour* and earlier newspaper reports by presenting animal suicide as an individual and ‘deliberate act of will’.^20^ Its reports generally involved dogs, believed to be the most sagacious and affectionate animals, which were ‘driven to this climax of despair’ following neglect, physical mistreatment, the death of their master, or even a sharp rebuke.^21^

But suicide was by no means considered the preserve of dogs and scorpions. In 1875, *The Animal World* reported a case of stag suicide on the south coast, with an accompanying illustration (see Plate 1), and criticized the presentation of blood sports as a noble pastime that was enjoyed equally by the hunting dogs and their quarry. ‘It is notable’, an editorial claimed, ‘that a wild stag, rather than be overtaken by its pursuers, will fall into the jaws of an awful death’.^22^ Once again, suicide was presented here as the last desperate act of a ‘notable and proud animal of high virtues and merits’. Cornered by ferocious dogs, the stag chose its fate. Like the endangered scorpion and the mistreated dog, it was ‘driven to desperation’.

**1.**
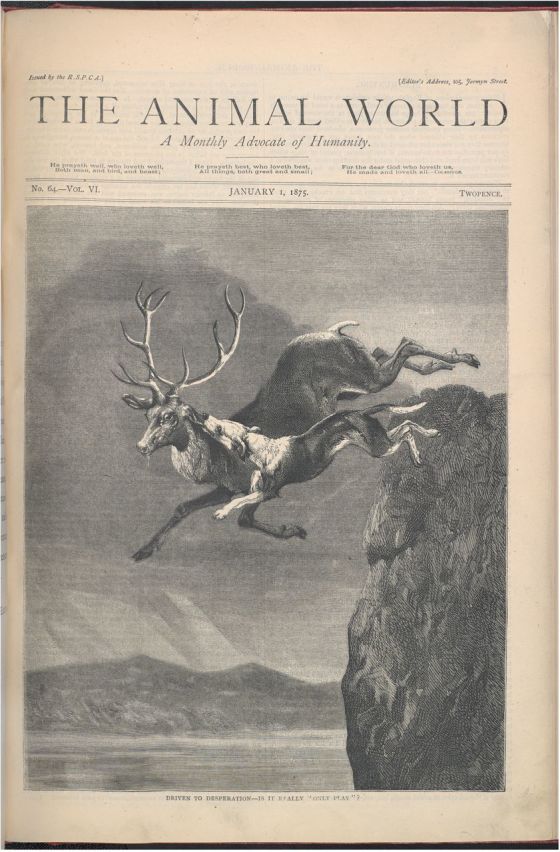
The front cover to a January 1875 edition of *The Animal World*, showing a reputed case of animal suicide on the south coast of Britain. Reproduced with permission of the British Library Board.

These anti-cruelty reports on intelligent and noble animals often cited scientific accounts of the apparent continuity between the animal and human mind (which themselves sometimes relied on popular reports of animal sagacity). The most famous example is Charles Darwin’s *The Descent of Man*, published in 1871, which argued that mind was subject to processes of evolution by natural selection, and there was therefore ‘no fundamental difference between man and animal in their mental faculties’. Any difference, Darwin concluded, was simply ‘one of degree and not of kind’.^23^ Indeed, in 1872, Francis Power Cobbe, the noted feminist, anti-vivisectionist and editor of *The Animal World*, wrote to Darwin claiming that reports of dog suicide offered striking evidence for mental continuity.^24^

Darwin did not respond to Cobbe’s letter and did not write directly on animal suicide, but his support for mental continuity drew on the strongest advocate of the reality of animal suicide in this period, the Scottish psychiatrist William Lauder Lindsay, superintendent of Murray’s Royal Asylum in Perthshire. When detailing links between the mental faculties of humans and animals in *The Descent of Man*, Darwin cited two articles that Lindsay had recently published in the *Journal of Mental Science*. Here, Lindsay claimed that instances of animal suicide demonstrated how ‘animals possess *mind of the same nature as man*; that there is no mental attribute peculiarly or characteristically human; and that there is, therefore, no mental distinction between man and other animals’.^25^

Lindsay provided a link between advocates of mental evolution and anti-cruelty campaigners. His support for mental continuity was motivated by a clear desire to inculcate greater sympathy for animals: to show that ‘if a dog or horse is not a man, he is at all events, in certain respects, *a brother*’.^26^ Lindsay was publicly renowned as a ‘sound and progressive alienist’, who rejected mechanical restraint of asylum patients, and he regularly appeared to extend into the animal kingdom the ethos of ‘moral management’ that was sweeping through the psychiatric profession in this period.^27^ Indeed, using the language of the influential reformist John Conolly, Lindsay claimed that human and animal suicide was ‘not the simple product of malady, but of malady aggravated by mismanagement’. Drawing from Conolly’s critique of forcible confinement, and extrapolating across species, he claimed that ‘when the law of kindness dictates man’s treatment of fellow animals — as it now regulates the management of his insane fellow man — destructive violence at least, and perhaps also suicidal despondency, will doubtless become less frequent’.^28^

At the same time, Lindsay also drew on the work of other nineteenth-century psychiatrists, who problematized the Romantic belief that suicide was a largely rational act in order to portray it more as a medical than a legal or moral problem.^29^ Figures such as James Cowles Prichard and, later, Henry Maudsley claimed that while victims of suicide intentionally killed themselves, their motivations were largely irrational and caused by ‘insane impulses’ that resulted from grief, jealousy, depression, physical illness or hereditary factors. Lindsay, too, presented animal suicide as predominantly caused by an ‘acute mania’ that stemmed from mistreatment, grief, jealousy, fear, captivity, ennui, old age, physical illness or brain disease.^30^ But framing suicide as the result of ‘acute mania’ did not diminish claims for animal intelligence. Lindsay firmly believed that while acute mania underpinned suicidal impulses by impairing the ‘instinctive love of life’ and attachment to others, the act itself remained ‘deliberate and intentional, the result of choice and consideration’.^31^ He regularly argued that suicidal animals, like people, displayed ‘the higher efforts of intellect’ in their often repeated attempts at suicide, comprehending clearly ‘the relation of cause and effect [and] the possibility of producing a certain end by the use of given means’.^32^

This latter claim was firmly rejected by Maudsley, who was widely regarded as Britain’s most influential psychiatrist during the late nineteenth century.^33^ In addition to achieving renown for developing Prichard’s ideas on ‘insane impulses’ during the 1870s, Maudsley had also written on the mental capacities of animals in two 1862 articles on ‘The Genesis of Mind’.^34^ In contrast to Lindsay and others, however, Maudsley in these articles dwelt on the mental limitations of animals. Maudsley argued that, while ‘higher’ animals such as dogs exhibited ‘kindly feeling and active sympathy’, they only possessed a rudimentary intelligence comparable to that of very small children or ‘congenital idiots’, and certainly far inferior to ‘ordinary human intelligence’.^35^

Maudsley applied these arguments directly to animal suicide in 1879, when he wrote an article in *Mind* dismissing reports that a sick dog had deliberately drowned itself. ‘It is quite possible’, he argued, ‘that an animal in a state of excitement or delirium from pain and illness may make a frantic rush that issues in its death, just as a human being may do; but this is quite a different thing to a distinctly conceived and deliberately perpetuated suicide’.^36^ Maudsley claimed that Lindsay and other believers in animal suicide were too uncritical of the correspondents and popular stories on which they based their conclusions, and had not ‘taken every pains to avoid the common fallacies of observation and inference’.^37^ He repeated these criticisms in an 1880 review of Lindsay’s two-volume *Mind in the Lower Animals in Health and Disease*, again claiming that Lindsay’s love of animals led him to assess uncritically sources of ‘dubious value’, so that ‘veracity seems to have been sacrificed, in some cases, to a spirit of romance’.^38^ Maudsley reiterated that while ‘insane impulses’ may well be shared across species, non-human animals lacked the mental ability to foresee that certain acts would result in self-destruction. He believed a more rigorous author than Lindsay would have instead listed supposed cases of deliberate animal suicide as accidental deaths owing to panic, fright or illness.^39^ ‘Suicide should hardly be considered as such’, he stated, ‘unless the animal has a distinct knowledge of death, and few animals indeed have a knowledge that death will follow certain actions, and will follow such actions to attain their end’.^40^

But by the time he wrote *Mind in the Lower Animals*, Lindsay was distancing himself from anecdotal and popular reports, which he admitted often ‘did not bear, or appear to bear, the stamp of truthfulness or authenticity’.^41^ He instead spent much of the two volumes arguing that better treatment for animals could only be secured through ‘dispassionate study’ of their mental endowments, and called for ‘comparative psychology’ to be established as a scientific field.^42^ Like contemporary figures such as Thomas Henry Huxley and Michael Foster, he believed that this should be founded on laboratory investigation, and that there was ‘no reason why the principle of *experiment* should not be applied to the investigation of the phenomena of Mind in the lower animals’.^43^ Lindsay believed that experimental work was crucial to ensuring better treatment for animals, by helping to dismantle ‘arbitrary and mischievous distinctions’ and engendering sympathy for creatures ‘to whom the application of the words *humanity* or *intellectuality* might be more fitly made’.^44^

Like Foster and Huxley, Lindsay also stressed that experiments would lead to a better understanding of human diseases, and then to therapies. He claimed that ‘experimental investigation on the lower animals has already been productive of contributions of the highest value to our knowledge of diseased or disordered function in Man’, and continued that further work ‘cannot fail to yield fruits of the most important kind alike to veterinary and medical science’.^45^ To Lindsay, the growing belief in the evolution of mental capabilities meant that psychological states in humans could never be fully understood without recourse to animals, and it ‘will only be when mind is studied in its most comprehensive aspect — not as confined to man, but as exhibited by the whole animal series, from its small beginnings to its highest development — that the necessary data will be collected for classifying in the spirit of modern science’.^46^

Lindsay devoted a whole chapter of *Mind in the Lower Animals* to outlining how ‘comparative psychology’ should be founded on ‘study by observation and experiment’.^47^ He argued the comparative psychologist should undertake experiments to ‘determine the true nature, relations and range’ of animal behaviour, under controlled and replicable conditions that ensured findings could not simply be dismissed as ‘*inferences* or *opinions*’.^48^ Most of the experiments Lindsay proposed involved prompting specific behaviour through actions and verbal cues, or watching how animals used tools to solve certain problems. However, he also detailed other experiments that were ‘cruel’ and should not be unnecessarily repeated, even though they might be useful for investigating animal intelligence. These included deception, such as replacing an animal’s eggs with stones, destroying nests and habitat, removing portions of the brain by vivisection, and encouraging ‘self-destruction’ in captive or distressed animals.^49^

Lindsay argued that these latter experiments were unnecessary since several investigators had already proved that animals ‘deliberately commit suicide’.^50^ To illustrate, he cited several cases in which researchers undertook experiments on animals to assess if, and under what conditions, they attempted suicide. Their animal of choice, the scorpion, reflected the continuing fascination with what the biologist Alfred Bourne called ‘the phenomena so graphically delineated by Byron’.^51^ In 1874, in an experiment that Lindsay recounted at length, one correspondent of *Nature* recounted how he had used a botanical lens to focus sunlight on a scorpion kept in a glass case, whereupon it ‘began to run hurriedly about the case, hissing and *spitting* in a fierce way’.^52^ During the fifth attempt of this experiment, when the scorpion apparently realized resistance was futile, it ‘turned up its tail and plunged the sting, quick as lightning, into its own back … sure enough in less than a minute life was quite extinct’.^53^ Writing to *Nature* in 1879, the Glasgow anatomist Allen Thomson claimed to have successfully replicated this experiment. ‘The effect of light’, he stated, produced ‘excitement amounting to despair, which causes the animal to kill itself’.^54^

Others contradicted Lindsay and called for more of these experiments, in order to shed greater light on the causes of suicide and the capacities of the animal mind. These included the jurist Wynn Westcott, who joined several writers in the 1870s and 1880s to argue that suicide should be perceived as a social and biological phenomenon which affected a growing proportion of the population and should be diagnosed and treated through medical and public policy.^55^ While he had dedicated himself to charting the statistical incidence of suicide among human populations, Westcott believed that animal experiments might prove critical in isolating causal factors. In his influential 1885 book, *Suicide: Its History, Literature, Jurisprudence, Causation and Prevention*, Westcott dedicated a whole chapter to animal suicide. He argued here that if experiments could be used to prove that animals did ‘kill themselves with the intention of ending their own lives’, it would have profound implications for the perception of suicide as caused by ‘heart-breaking grief of mind, or intolerable pain of body’.^56^ He then listed a number of favourable examples, including scorpion experiments, yet claimed that more work was needed to ‘close the controversy as to whether animals ever do, or do not commit suicide’.^57^

Westcott’s calls for more research were echoed by the comparative psychologist George Romanes, whose 1882 book *Animal Intelligence* was, like *Mind in the Lower Animals*, a compendium of stories highlighting the mental capabilities of many animals, with a view to establishing general principles for a theory of mental evolution.^58^ Although Romanes considered accounts of self-stinging scorpions to be ‘remarkable’, his belief that comparative psychology should have a firm empirical basis led him to demand even ‘further corroboration before we should be justified in accepting [them] unreservedly’.^59^

The call for ‘corroboration’ was answered the following year, when the experimental psychologist Conwy Lloyd Morgan, then living in South Africa, submitted a long letter to *Nature* detailing his own experiments on scorpions. Morgan had learnt laboratory methods at Huxley’s Royal School of Mines, in South Kensington, during the late 1870s. In addition to Huxley’s belief that biology could only be advanced through experimentation, Morgan also inherited his former tutor’s interest in the relation between consciousness and physical processes, and his disdain for the anthropomorphism of figures such as Romanes.^60^ As his letter to *Nature* made clear, Morgan shared Huxley’s belief that animal behaviour, including behaviour that was supposedly the result of conscious thought, should instead be explained in terms of simple reflexes.^61^ Morgan recounted how he sought to prove this by designing a series of experiments ‘sufficiently barbarous to induce any scorpion with the slightest suicidal tendency to find relief in self-destruction’.^62^ These experiments involved condensing sunbeams on to scorpions’ backs, burning them with acid and alcohol, surrounding them with fire, heating them in a bottle, and exposing them to electrical shocks and other ‘general and exasperating sources of worry’.^63^

Morgan argued that while scorpions clearly struck at their own backs, this should be interpreted as an instinctive effort ‘to remove the source of irritation’, and not as a conscious effort at suicide.^64^ Here, he advanced the argument that formed the core of his famed ‘canon’ during the 1890s: that objectivity and anthropomorphism were mutually exclusive, and that no animal activity should be interpreted as the outcome of a higher mental faculty if it could be reasonably interpreted as the outcome of one lower in the psychological scale, such as instinct or trial-and-error learning.^65^ Taking a thinly veiled swipe at the anthropomorphic work of Romanes, Lindsay and others, Morgan concluded that instinctive actions of the scorpion had previously been ‘put down by those not accustomed to accurate observation as attempts at self-destruction’.^66^

As we have seen, this argument had previously been made by Henry Maudsley, who claimed that an individual ‘sees only in any matter that which he brings with him the faculty to see’, and ventured that untrained observers and animal-lovers were misinterpreting accidental deaths as evidence of deliberate suicide.^67^ We should note, however, that Maudsley and Morgan had differing reasons for rejecting animal suicide. Maudsley’s stance reflected the views laid out in his work on ‘The Genesis of Mind’, where he sought to reassert an anthropocentric world view in the face of growing claims for animal intelligence. Throughout these articles, Maudsley regularly asserted that the ‘civilisation of today is greatly superior, in its practical morality, to the moral condition of the world at any other period’. As such, he refused to countenance that the minds of ‘cultivated’ human races were in any way comparable to those of lower animals.^68^ In doing so, Maudsley also portrayed the human mind as the only appropriate object of study for psychiatrists: since it was ‘so far exalted in its development above the animal mind’ and was ‘subject to the possibility of much greater degradation’. To Maudsley, ‘even the madness of man, then, declares his superiority’.^69^

Morgan’s dismissal of animal suicide, meanwhile, reflected the late nineteenth-century belief that cautious and detached readings of natural phenomena were crucial to the construction of a ‘scientific self’. As Greg Radick has shown, for a new generation of researchers seeking to establish comparative psychology as a respectable academic discipline, Morgan’s firm anti-anthropomorphic stance served as a badge of professionalism.^70^ In psychology and other disciplines, rejecting conscious thought in favour of automatic response separated the objective scientists from their subjects, and transformed the animal into a predictable and productive experimental tool.^71^ This professionalizing tactic helped displace the belief that animals could consciously end their own lives with the view that they were driven by the instinct of self-preservation. It was man’s capacity to overcome this instinct that separated him from the animal world.

## III

### SUICIDE, MODERNITY AND THE LEMMING

From the late nineteenth century there emerged two general, and largely antithetical, approaches to the study of human suicide that would dominate the field into the mid twentieth century. The first was the sociological or ecological, focused on identifying patterns in the statistics of reported suicides in the general population and linking them to factors such as population density, and social isolation and mobility. The second, most prominent among psychiatrists and psychologists, was focused on case histories, seeking to understand the precipitating, and often unconscious, factors that drove an individual to commit a self-destructive act. While animals were generally perceived as driven by the instincts of self-preservation, as we shall see, ideas of natural laws and inherent drives determining processes of self-destruction did leave space for the consideration of animal suicide. Further, with an increasing focus on the collective and unconscious elements of suicidal behaviour, attention turned to animals that were unwittingly driven to destruction: to shoals of fish dashing themselves on boat hulls, whales beaching themselves on the shore, or the hordes of lemmings known periodically to march across the Norwegian planes to perish in the sea.

The most influential exponent of the first approach was undoubtedly Durkheim. His analysis of the problem was closely tied to his struggle with the discipline of psychology and his promotion of the rules of the sociological method — a naturalistic methodology that identified ‘realities external to the individual … as definite and substantial as those of the psychologist or biologist’.^72^ In *Suicide*, a most private and individualistic act was explained sociologically. He first defined suicide as an act that the individual knew with certainty to be fatal. He excluded people with impaired reason and non-human animals, and limited its frequency in children and primitive populations. In the animal, any ‘void created by existence’ was purely physical, a matter of material resources, and when ‘filled, the animal, satisfied, asks nothing further. Its power of reflection is not sufficiently developed to imagine other ends than those implicit in its physical nature’.^73^ As human needs became less dependent on the physical body, the less easily were they satisfied. Second, he understood the various individual motives for suicide as driven, in turn, by broader and more fundamental social currents. Conscious acts were determined by unconscious forces to be studied by the sociologist. The breakdown in traditional forms of social solidarity and the rise of egoistic individualism were reflected in the rising rates of suicide in Western nations.

Peter Hamilton identifies Durkheim’s work as steeped in naturalistic metaphors: populations had their own suicide rates, determined, for Durkheim, by ‘real living active forces’ independent of the individual.^74^ This deterministic tendency was shared by other contemporary moral statisticians. Durkheim had drawn heavily on the work of Enrico Morselli, professor of psychological medicine at the Royal University of Turin and physician-in-chief to the Royal Asylum for the Insane. For Morselli, like Durkheim, suicide was a ‘voluntary human act’.^75^ And yet, it could only be understood through the statistical study of the social forces that motivated the act: ‘The old philosophy of individualism had given to suicide the character of liberty and spontaneity, but now it became necessary to study it no longer as the expression of individual and independent faculties, but certainly as a social phenomenon allied with all the other racial forces’.^76^ The motives of the collective outweighed the personal. Morselli argued that without the aid of statistics, motives were ‘not apparent; there are other, more secret causes whose existence and influence elude even the suicide himself, because they act on him unconsciously’.^77^ As civilized as human beings had become, they were still subject to the laws of nature. While the ‘savage’, being closer to the animal, rarely resorted to suicide unless through fanaticism or under the ‘stress of hunger’, with increased population density, urbanization, organization and individualism came new ‘psychical (cerebral) needs’, and increased rates of suicide.^78^ Just as disease, sterility and murder removed the weak among animals and primitive men, suicide functioned as a ‘tribute’ in advanced society, removing a certain number of individuals in accordance with Darwinian and Malthusian principles.^79^

S. A. K. Strahan, psychiatrist and fellow of the Royal Statistical Society and the Medico-Psychological Association, was even more explicit in interpreting suicide as a process of natural law. He first posited self-preservation as ‘the first law of nature’.^80^ Any animal in which this instinct was weak or lacking was thereby unfit, and ‘the creature must go’.^81^ The ‘savage’ also, ‘in common with the wild animals, exists under conditions more or less approaching the natural’,^82^ while the ‘idiot’ rarely committed suicide, being ‘led exactly like the beast of the field’.^83^ Strahan drew upon the work of Morselli and turned to heredity for an explanation for the increase in suicide in civilized nations. While carriers of suicidal impulses were eradicated in nature, they, like those of numerous other pathologies and degeneracies, were sustained in the modern world and allowed to propagate their kind, until ‘an active, all-consuming desire for death is developed’.^84^

John C. Weaver identifies Durkheim, Morselli and Strahan as leading figures in a group he designates as determinists.^85^ The regularity of suicide statistics and their steady increase in civilized societies revealed a general law. Suicide was no longer merely an issue of free will and moral censure; a larger social or natural law determined that a certain number of individuals would end their own lives. In the work of Morselli and Strahan, in particular, human beings were placed within nature, subject to the same processes of struggle, strife, degeneration and decay that removed a certain proportion of the population, and yet they were also differentiated from it through their capacity for self-destruction that grew with the advance of civilization, serving as the means by which the *human* population limited its numbers. For those focused on linking social and biological laws of population dynamics, the fact that suicide rates seemed to increase with population size, density and geographic mobility provided a space for considering more general processes of destructive behaviour in the natural world.^86^

Raymond Pearl, based at Johns Hopkins and considered the leading population biologist in the United States, was determined to bring the social and biological sciences together through statistical study.^87^ In order to achieve this he turned to the lemming, which had become of interest to biologists since travellers reported their behaviour in the late nineteenth century. This was because the lemming’s actions seemed so destructive, challenging, as one writer put it, ‘the whole doctrine of the preservation of the species’.^88^ Following an explosion in numbers every few years, masses of lemmings were seen to travel down from their mountain homes to the coast.^89^ Undeterred by the sea, they swam out until they drowned, littering the beaches with their corpses. When marching, they did not seem to move aside for any obstacle, as if, one writer described, they were ‘impelled by some strange power’.^90^ The lemming was described as indifferent to danger and death, often responding to approach with rage, frenzied attack and even a ‘poisonous’ bite.^91^ The saying ‘angry as a lemming’ was common in Norway and, no doubt to the consternation (or amusement) of those visiting Scandinavia in a ‘lemming year’, the animals were believed to get so enraged they were liable to ‘explode’.^92^ With such associations — of mass movement, aggression and volatility — it is perhaps unsurprising that military metaphors proved popular; a report by a Mr Brooke in 1823 described their crossing of a body of water in terms of an ‘army’ forming a ‘complete pontoon bridge’.^93^

Lauder Lindsey had suggested that the march of the lemming ‘armies’ could be seen in terms of an ‘epidemic morbid impulse, leading to epidemic suicide’, but admitted that he could not explain ‘the object or cause’.^94^ For Pearl, there was a clear Malthusian explanation. Lemming population cycles could be mapped on to the growth and decline of races and civilizations. A fundamental biological law, he argued, governed both. For Pearl, the ‘mass suicide’ of the lemming was a particularly brutal example of the processes by which a species regulated its numbers to ensure its long-term survival. While he argued in 1937 that one could not envisage the same ‘watery grave’ for humanity, he nevertheless believed that humans were being driven to war, and thus to ‘commit suicide’, by the same fundamental biological forces of growth and decline.^95^ Pearl’s Malthusian approach was shared by an emerging discipline of ecology. Charles Elton ensured that the lemming cycle would be central to the work of the Animal Population Bureau at Oxford, the centre of the growing field of animal ecology in Britain.^96^ For Elton, overpopulation itself was the cause of the lemmings’ migration and their subsequent demise.^97^ This he described as ‘a rather tragic procession of refugees, with all the obsessed behaviour of the unwanted stranger in a populous land, going blindly on to various deaths’.^98^

Throughout the twentieth century, the lemming increasingly came to symbolise man’s capacity for self-destruction. Its popularity increased with the destructiveness of the world wars, as Pearl’s work shows. Particularly prevalent was its use as a symbol of Nazi Germany’s collective, self-destructive urge for *lebensraum*, what Northrop Frye described as Hitler’s ‘lemming march’.^99^ For Sir Lewis Namier, Hitler had made use of the most fundamental element in politics — the ‘primal horde’ — and ‘there can be no free will in the thinking and actions of the masses, any more than in the revolutions of planets, in the migrations of birds, and in the plunging of hordes of lemmings into the sea’.^100^ Through the lemming, the human being was presented as a destructive animal. Yet this was not destruction motivated by passion; in the words of the psychiatrist Erich Fromm, it was that of the ‘totally alienated’, the ‘automaton’, not the ‘destroyer’.^101^

Such interpretations were no doubt reinforced by *White Wilderness,* a Disney nature documentary from 1958, often mistakenly regarded as the source of the lemming suicide myth, in which a large number of lemmings were forcibly marched to their deaths in Alberta. The narrator described them as gripped by a ‘compulsion’, an ‘unreasoning hysteria’, as ‘victims of an obsession, a one tracked thought’. The image of thousands of identical rodents following a ‘leader’ to their destruction was here, perhaps, associated with the dangers of communism; but it was just as commonly related to the banality of party politics and the conformity of consumer culture.

The lemming was, therefore, not an example of an individual animal *wilfully* ending its life in defiance, anger or grief, as in the case of the Romantic scorpion or the Victorian dog; this was a suicide of the unconscious mind, the machine, the unthinking mass, species or system. While, in the nineteenth century, suicide had been a means of attributing emotion, mind, intelligence and individuality to the animal, in the twentieth century its role was reversed — a means of questioning the independent intellectual faculty of the modern human. Indeed, for Fromm: ‘In the nineteenth century inhumanity meant cruelty; in the twentieth century it means schizoid self-alienation’.^102^

Psychiatrists and psychologists were also much taken by the lemming as a means of illustrating the alienation of modern people.^103^ For the psychologist and concentration camp survivor Bruno Bettelheim, the behaviour of the lemming was not only characterized by aggression, but reflected fatalism: ‘The unique feature of the extermination camps is not that the Germans exterminated millions of people … What was new, unique, terrifying, was that millions, like lemmings, marched themselves to their own death. This is what is incredible; this we must come to understand’.^104^

Bettelheim believed that the behaviour of individuals in the camps had provided him with an understanding of the human condition in a mass society. With industrialization, urbanization and depersonalization there emerged ever greater threats to individual autonomy and ever increasing degrees of alienation. In the camps, thought and consciousness had been sacrificed for physical preservation. Individuals ‘stopped acting on their own’, becoming withdrawn, fatalistic, childlike. Psychic death soon led to physical death as the individual lost the struggle for life. ‘The prisoners lived, like children, only in the immediate present; they lost the feeling for the sequence of time, they became unable to plan for the future or to give up immediate pleasure satisfactions to gain greater ones in the near future. They were unable to establish durable object-relations’.^105^

The same processes that reduced humanity to a mass of automatons or ‘walking corpses’, disconnected from the world around them, Bettelheim found in autism, the disease of civilization that was to occupy him for the rest of his life. Autism, often defined as childhood schizophrenia, was an individual’s response to feeling totally overpowered. In order to prevent complete annihilation, the individual committed a kind of ‘psychic suicide’, withdrawing from human contact and existing in a silent, dream-like state, oblivious to danger yet prone to momentary bouts of extreme anger.

For Bettelheim, however, the suicidal lemming was a metaphor, not an analogy. Indeed, it was the fact that the lemming’s behaviour seemed so bizarre and incongruous that made it such a powerful rhetorical tool; not so much a means of uniting humanity with the natural world, but dividing them from it. When it came to studying the actual causes of suicide, psychologists and psychiatrists were drawn to psychoanalytic techniques, and duly promoted the individual case history over statistical patterns and laws of population growth and decline. Two of the leading figures in the psychoanalytic school of suicidology, Karl A. Menninger and Gregory Zilboorg, were particularly dismissive of statistical approaches. Menninger described statistical analyses as ‘barren’, and his influential book, *Man against Himself*, had little on social factors.^106^ Zilboorg criticized the methodology of sociologists and moral statisticians: ‘Statistical data on suicides … deserve little if any credence … since all too many suicides are not reported as such’.^107^

It was also the case that ‘suicide attempts, no matter how serious, never find their way into the tables of vital statistics’.^108^ Both Menninger and Zilboorg were active in broadening the definition of suicide to encompass the attempted or ‘partial’. This allowed them to overcome a serious methodological obstacle to psychoanalytic study. The psychoanalyst relies on a detailed examination of the individual’s life history. This is, of course, not possible with the suicide unless the individual also happened to be under long-term psychiatric examination. It does become possible, however, if one includes those who attempt, or even think about, suicide and those who self-harm.^109^ The focus was broadened to address a more general self-destructive impulse, and Menninger included a range of behaviours within the category of suicidal behaviour, such as self-starvation, dehydration and the refusal of medical treatment (organic), accidents, dangerous sports and smoking (chronic suicide), and self-mutilation (partial and focal suicide). He drew upon Freud’s notion of the death instinct or drive, which, in direct contrast to the pleasure principle, was a conservative force that had evolved as a means of alleviating tension and coping with trauma — ‘an urge in organic life to restore an earlier state of things’,^110^ assuring, ultimately, ‘that the organism shall follow its own path to death’.^111^ For Menninger, this innate, unconscious and destructive drive was common to all and, when not effectively balanced by the instinct of self-preservation, and when aggressive and destructive impulses found no outlet, it underlay a variety of self-destructive behaviours. Self-harming, or partial suicide, was, in turn, a final attempt at self-preservation.^112^ Recalling Bettelheim, he argued that localized self-destruction was a means of averting total suicide. While Menninger did not focus directly on the possibility of animal suicide, the universality of the self-destructive drive certainly created a space for its consideration, and his work does contain a number of examples, from mink chewing off their limbs when trapped and a monkey that self-mutilated when emotionally conflicted, to helpless rats that gave up their struggle for life and succumbed to death.^113^

Zilboorg, a psychoanalyst based in New York City, chaired a short-lived Committee for the Study of Suicide, established in 1936. The Committee supported research on suicide among children and the ‘primitive races’, such as the Mohave.^114^ While Zilboorg considered the idea of a death instinct to lack explanatory power,^115^ he similarly described suicide in terms of a biological instinct that ‘appears to be a real elemental psychic force, universal in nature and apparently confined not alone to human beings’.^116^ He outlined some of the various dynamic motivational factors at work, arguing that ‘… suicide is far from being the monopoly of civilization’.^117^ Not only was it as common among primitive populations, but ‘animals, too — for instance, dogs or monkeys — on occasion refuse food and die when mistreated’.^118^ Zilboorg argued that psychoanalysts needed to understand how such an instinct could have evolved, and suggested that, just as the body’s response to physical injury could end in the death of the organism, when the mind was fearful and frustrated by its inability to master reality it projected fantasies and delusions to the point that it ‘saved’ itself through the paradoxical self-assertion of self-imposed death.^119^ The idea that suicide involved the exercise of free will and rationality was, for Zilboorg, ‘medieval’ and left over from ‘theological age’.^120^ It was critical, he argued, to piece together the various unconscious and instinctive motivations to classify and formulate a typology of suicidal behaviours.

Thus, in their different ways, the ecological and psychoanalytic approaches advanced the secular study of suicidal behaviour, one promoting social and biological factors external to the individual, and the other, internal and unconscious factors. Both complicated the conception of suicide as an intentional act involving the exercise of free will and rational motivations. However, as Zilboorg observed, ‘a truly scientific psychology of suicide is still wanting’.^121^ He believed, as did many by the mid twentieth century, that scientific progress depended on greater synthesis between the study of psychological motivations and that of social conditions.^122^ He also pushed for closer relations between psychoanalysis and the natural sciences, biology in particular.^123^ The stage was set for a reconsideration of the role of the animal as a means of understanding suicidal behaviour. As we shall see in the next section, the experimental laboratory was emerging as an important means of bringing the social and the psychological approaches together, identifying how various instinctive drives and external determinants combined to result in self-destructive behaviour.

## IV

### EXPLAINING THE SUICIDAL LEMMING: STRESSED RATS, NEUROTIC CATS AND DEPRESSED MONKEYS

The lemming’s behaviour had become a recurring motif for modernity; yet how, exactly, did one explain its ‘suicidal routine’?^124^ Seeking to understand the processes that determined animal population dynamics, many, Pearl included, turned to the laboratory. Of particular importance was the work of a group of animal ecologists employed on the Rodent Ecology Project at the Johns Hopkins School of Public Health. The project, established in 1942, had been devising ecological methods of rat control.^125^ By restricting the access of the animals to food, water and nesting sites, competition for resources would be increased and, with it, violence, reproductive dysfunction and death.^126^ Critical to this method was the work of one of the project researchers, John B. Calhoun, whose approach had been inspired by Pearl’s studies of closed laboratory populations, yet was increasingly informed by the ideas of psychologists and psychiatrists.^127^ Seeking to understand the social behaviour of the rat, in 1947 Calhoun kept a small number in a quarter-acre pen behind his house in Towson Maryland and, later, once employed by the National Institute of Mental Health (NIMH) from 1954, in a laboratory in a converted barn.^128^ Providing the animals with unlimited supplies of food, water and nesting materials, he described his rodent universe as a ‘rat utopia’. With the subsequent increase in numbers in a confined space, the pens soon heaved with animals, and utopia rapidly descended into ‘hell’.^129^ Dominant males became aggressive and hypersexual marauders, attacking females, juveniles and less active males, while females stopped caring for their young, resulting in an infant mortality of 96 per cent. One, ‘ultimate’, pathology captured his imagination. In a series of later experiments in a ‘mouse paradise’, there emerged a class that no longer competed for territory, status, food or mates, but huddled together in a motionless, silent mass, eating and drinking in unison, and prone to sudden bursts of extreme violence. At the highest levels of density, mice at the very bottom of the social hierarchy had sacrificed their individual identities as a means of physical preservation; they had stopped being mice, existing only as ‘hollow shells’, ‘somnambulists’, ‘zombies’. Calhoun described this pathology as ‘social autism’, the autism of the group or mass. When entering into his experimental universe to be photographed for *Life* magazine, females moved in unison, following his feet around the pen (see Plate 2). They were fearless and obsessive, the result of their infantile psychological state. Unable to breed, the experimental population tailed off to extinction.

**2.**
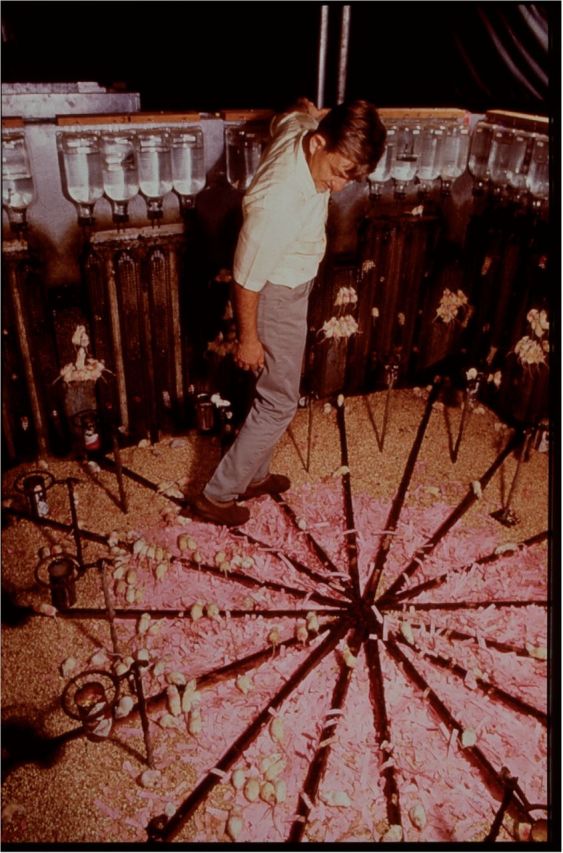
John B. Calhoun entering an experimental mouse ‘universe'. From John B. Calhoun Papers. Image courtesy of the National Library of Medicine.

Prior to his employment at the NIMH, Calhoun had been briefly employed, from 1951 to 1954, at the Walter Reed Army Medical Center. He served as a member of the neuropsychiatry division, the military being curious as to the possible connections between the mass behaviour of human beings and that of lemmings.^130^ It was not the mass migration of the animals but their destruction that would prove his most enduring interest. Crucial was the concept of *stress*, as suggested by the ecologist and physiologist John J. Christian, Calhoun’s colleague at Johns Hopkins. Christian was the first to apply the work of Hans Selye to animal ecology, arguing that the social and psychological breakdown witnessed in Calhoun’s crowded pens had physiological causes and consequences.^131^ Selye had applied acute, non-specific nocuous agents to rodent bodies: extreme cold, surgical injury, excessive exercise and injections of sub-lethal doses of various drugs. All had produced a typical ‘general adaptation syndrome’, the body responding to ‘stress agents’, either physical or behavioural, through the hypothalamus, enabling the adrenal glands to release hormones to maintain equilibrium. Selye had, in turn, built upon Walter Cannon’s physiological principles of homeostasis: emotions of rage and fear, necessary for ‘fight or flight’, could, if severe, have destructive effects on the nervous system. For Selye, it seemed that under conditions of constant stress, metabolic changes allowing for reaction, resistance and the maintenance of equilibrium became ‘diseases of adaptation’, increasing mortality through adrenal hypertrophy, hypertension, ulcers, and kidney and heart disease.

For Calhoun and his associates, errant behaviours and their resultant stress-related illnesses served to dampen population growth, ensuring that animals did not outstrip their means of subsistence. Homeostasis at the level of the body functioned, therefore, at the level of the group and the species; while stress could be destructive for the individual, psychopathological behaviour had important, and necessary, ecological consequences. For Christian, crowding stress not only provided a solution to Baltimore’s rat problem, but also to the lemming question. Dennis Chitty, of Oxford’s Bureau of Animal Population, agreed. Under the direction of Charles Elton, Chitty was also engaged in a rodent control project in Britain. Instigating his own studies of crowding stress in the vole, he identified the same excessive violence, sexual deviance, withdrawal and increases in morbidity and mortality. There existed, he declared, ‘a general law that all species are capable of regulating their own population densities without either destroying the food sources … or depending on enemies or climatic accidents’.^132^ The mass ‘suicide’ of the lemming was not the consequence of a purposeful march towards the sea; it was the inevitable destruction, through stress-related behaviours and illnesses, of a proportion of a population as an ecological system returned to a condition of stable equilibrium. The lemming was more than a mere metaphor: it was mentally and physically ill, a neurotic, its personality shredded and disintegrated by conditions of high density. Biologically predisposed to be a ‘rugged individualist’, to live among the few not the many, it suffered when, like human beings, it was forced to live among the crowd.^133^

There is, however, considerable and persistent slippage in Calhoun’s writing between the rhetorical and scientific uses of the lemming. In a paper that was presented to the United States Congress in 1971, Calhoun made the connection between the suicidal lemming and the future of humanity because it was powerful and dramatic. Lemmings, like human beings, seemed determined to stand apart from the laws of the natural world, to disrupt the so-called ‘balance’ of nature.^134^ It allowed him to draw attention to a fundamental problem facing civilization — too many people in too little space. The result was already evident in the city: suicide, murder, rape, self-harm and, above all, the ‘second death’, the death of the mind: ‘Lemmings are lemmings … Mice are lemmings. Are there lemmings in our metropolitan tundras — silent shadows of the selves they might have been ready to follow in unquestioning masses any flickering figment on a glassy screen? Are they ready to bring civilization to suicide?’^135^

The lemming population found relief from its intolerable situation by means of its ‘suicidal’ behaviour, allowing for its ‘rebirth’. Human beings, however, were trapped on ‘Spaceship Earth’, just as Calhoun’s mice were trapped in their crowded pens: ‘there can be no escape if things go awry’.^136^ Further population increase, Calhoun surmised, could have such devastating effects on humanity that there would be no ‘rebirth’. This led Calhoun to reflect: was the lemming an animal or was it an idea? If the latter, were people more like lemmings than lemmings?^137^

Yet, at a scientific level, the case of the lemming emphasized the importance of the animal laboratory for the understanding of a huge range of behavioural anomalies and mental and physical illnesses. In the work of scientists, the lemming had a dual role: it was, owing to its cultural resonance, a means of attracting attention to the effect of the environment on behaviour and the perilous advance of human civilization; yet, underlying the lemmings’ apparent urges for self-destruction were the real processes of stress, processes that could be understood through animal models. It was through the animal that suicide could be understood not as an individualistic act of free will, but as the consequence of social and biological forces. In the growing field of environmental stress, animal research stimulated the study of the ill-effects of crowding among humans, combining ecological approaches with case histories.^138^ The Edinburgh psychiatric epidemiologist D. H. Stott, for example, argued that cases of ‘pathological mothering’,^139^ self-mutilation^140^ and delinquency could be seen as natural ‘genetic provisions for lethal defect’ activated among those at the bottom of the social hierarchy.^141^ The law of the lemming was active in the city: ‘The young animals in the course of their wandering often show a disregard for their own safety analogous to the inconsequent behavior typical of many delinquents’.^142^ Such ‘suicidal’ behaviours had evolved because they had purpose or survival value for the population: ‘limiting the numbers of a species to what can be supported by the available food resource’.^143^ In an analysis of prisons, medical psychologists identified correlations between physical space and ‘increased suicides, psychiatric commitments, disciplinary infractions, violent deaths, and deaths due to natural causes’.^144^ For Ivor Mills, professor of medicine at Cambridge and a leading scientist of stress in Britain, people were ‘human lemmings’: individuals ‘rushing madly to or from work’, driven into aggressive competition by overpopulation and overcrowding. ‘There has’, he reflected, ‘been a rapidly rising incidence of “attempted suicide” in this country’.^145^

Calhoun’s research on population dynamics dovetailed with emerging fields of experimental psychiatry and psychobiology in the United States. As Zilboorg noted, experimenters had come across cases of extremely disturbed behaviours in their laboratories.^146^ By building upon such cases, psychiatrists such as Jules H. Masserman at Yale sought to bridge what he described as the ‘chasm’ between psychoanalysis the study of ‘lower’ forms of life.^147^ For Masserman, human beings differed from other chordates only with regard to the degree of versatility provided by their ‘many technics of adaptation’.^148^ The underlying causes of mental disorders were the same. Thus, he argued, in the controlled environment of the laboratory and by following animals through time, it was possible to provide a better understanding of affective disorders. With regard to Freud’s death instinct, they could examine if ‘ostensibly self-punitive or self-destructive behavior occasionally observed in animals’, and in Norwegian lemmings in particular, were ‘based on deviant individual experiences without primal atavistic urges’.^149^ While Masserman focused his attention on the alcoholic behaviour of neurotic cats, rather than suicide per se, he was pioneering in his attempts to provide the theories of psychoanalysis with a rigorous and objective scientific basis through the animal laboratory.

As the field of experimental psychiatry developed through the mid twentieth century, its practitioners refined the standards of validity regarding the animal model. They increasingly emphasized heuristic value over behavioural similarity; that an animal model could mirror exactly a specific behavioural disorder in humans was considered a ‘utopian goal’.^150^ The importance of ‘face validity’ (surface equivalence) was not as great as ‘construct validity’ (functional equivalence). Influenced by the growing number of ethological and ecological studies of animal behaviour, psychiatrists and psychologists were seeking models of psychopathology that relied less on the use of artificial methods, such as electric shocks, to replicate a human disorder, as in Masserman’s studies, and more on the manipulation of meaningful functional relationships between animals in their social and physical environments. For example, the psychiatrist William T. McKinney, a leading figure in delineating standards of validity in modelling, joined the psychologist Harry Harlow at the University of Wisconsin–Madison in the late 1960s to study the psychopathological effects of isolation among rhesus monkeys. McKinney argued that the study of isolation among animals was of value ‘in its own right to better understand the underlying mechanisms associated with separation reactions. It is not necessary and even dangerous, perhaps, to make a priori assumptions about clinical labels that might apply to separation, to social isolation or other situations’.^151^ Such clinical labels, and their related etiologies, were often problematic in any case, and animal studies could ‘help clarify in a more systematic way some parameters of the human syndromes’.^152^ Isolation from mothers and peers functioned, just as Calhoun’s crowded housing, as an experimental system that allowed for the study of the underlying causes and mechanisms of a wide range of psychopathologies, including various forms of self-destructive behaviour.^153^

The early life experiences of Harlow’s animals were identified as critical to their future psychological health. Among the isolated monkeys, some would withdraw and become severely depressed, displaying ‘autistic self-clutching and rocking’, and refusing food to the point of dying from ‘emotional anorexia’^154^ — the consequence of ‘psychological death produced by social loss’.^155^ Others resorted to violence, expressing ‘suicidal aggression toward adults’, or ‘inwardly toward the animal’s own body’.^156^ In a series of studies that built upon Harlow’s work, the psychiatrist Ivor Jones described how: ‘The confined macaque inflicts severe injury with its teeth and claws, gashing limbs, trunk, and scrotum and making deep bites to accessible sites on its own body’.^157^ Such violent behaviours, often resulting in the death of the animal, were again described in the context of a ‘homeostatic mechanism’: venting aggression on the self or others restored the degree of arousal to ‘tolerable limits’,^158^ and served as ‘a way to self-regulate at times of stress’.^159^

Psychiatrists such as Bettelheim drew directly on such experiments, focusing more fully on the effects of isolation in various dehumanizing regimes and destructive environments.^160^ Karl Menninger combined the concept of stress with psychoanalysis as it gave physiological support to ideas of homeostatic regulation in psychological processes. He noted how the behaviour of experimental animals was ‘analogous’ to the ‘catastrophic breakdown’ of human patients under extreme stress.^161^ The influential British psychiatrist John Bowlby combined the work of Harlow with that of ethologists such as Konrad Lorenz, praising them for having finally provided ‘an adequate scientific framework’ which could identify why the disruption of affectional bonds could cause an individual to be ‘crushed by grief and die of a broken heart’ or ‘do things that are foolish or dangerous to himself and others’.^162^ He also focused on aggression and frustration, suggesting correlations between self-injury among animals and self-injurious and suicidal states in human beings. Ivor Jones not only posited associations between the behaviour of ‘macaques in confinement’ and socially deprived children — rubbing, biting, scratching, head-banging and hair-pulling — but between the high rates of self-mutilation among adult prisoners and the behaviour of caged animals.^163^ While there was much to differentiate between a laboratory monkey and a lemming in the wild, Jones argued that animals experienced isolation within, and because of, the crowd.^164^

While Jones noted that it was commonly assumed that ‘thought’ initiated the act of self-injury among humans, ‘the order may in fact be reversed, thought being used to elaborate and transform, rather than initiate’.^165^ For Jones, in common with psychoanalysts, self-injury and suicide existed on the same continuum, the former a means of understanding the latter. Thus, while many assumed that it was only humans that committed suicide by virtue of being able to visualise and arrange their own deaths:

... this formulation implies that the processes leading to suicide are rational, which may be untrue: depression in most suicides probably impairs the capacity for rational thought while at the same time inducing suicide impulses. We suggest that suicide may be a uniquely human attribute only because our definition of it makes it so; in other words, if suicide were to be defined as a destructive act inflicted on the self leading to death, then animal analogies do exist.^166^

Humans did, of course, have many more techniques for self-destruction at their disposal. However, the non-human animal was seen to provide a means of understanding the underlying psychobiological mechanisms and processes which, in combination with social and ecological factors, determined suicidal behaviour in man. Depression, hopelessness and self-harming as release from stress and tension were seen to underlie suicidal acts, as scientists increasingly pushed for the understanding of suicide as a manifestation of a broader class of behaviours.^167^ For this reason, the use of animal models in the study of self-destructive behaviour continues apace in psychology and psychiatry.^168^ Interest in self-destructive animals has also been further encouraged by the development of the fields of sociobiology and evolutionary psychology, with E. O. Wilson beginning (and concluding) his controversial tome with reference to Albert Camus’ famous declaration: ‘There is but one truly serious philosophical question and that is suicide’.^169^ Sharing the approach of animal ecologists, sociobiologists aim to explain the evolutionary purpose of self-destructive behaviour. While, much like the scorpion, the lemming is no longer seen as directly analogous to a human suicide in scientific literature,^170^ the study of self-destructive behaviour remains focused on stress as a critical psycho-physiological mechanism that functions in both individuals and populations, human and non-human. Concomitantly, and in contrast to the ideas of the nineteenth century, those who object to the concept of the animal suicide do so not because it concedes intention, will and reflective action to the non-human animal, but because it is seen to deny them to the human. As one of the leading suicidologists to focus on understanding individual and personal motives, Edwin S. Shneidman declared suicide needed to be analysed in terms of ‘conscious intention’: ‘We shall not be concerned at all with migrating lemmings or mourning dogs’.^171^

## V

### CONCLUSION

The self-destructive animal is central to our understanding of the nature of suicide. Even when used as a means of criticizing the extremes of anthropomorphism, it performs a crucial social and philosophical function.^172^ When people reject the possibility of an animal committing suicide, they reserve not only the act itself for humans, but many traits that enable it — emotion, intelligence, mind and consciousness. As a distinctly ‘human privilege’, suicide becomes constitutive of the human, as Jean Baechler argues: ‘Not only does suicide presuppose a conscious being, it is also present as a potential within every conscious being. That is why neither animals, very young children, nor sick people whose mental faculties have been destroyed commit suicide’.^173^

The prevailing understanding of suicide as a uniquely human act is dependent on our understanding of the non-human — commonly viewed, in the words of Henri Bergson, as a creature ‘clinging to life’, simply ‘carried along by its impetus’.^174^ Yet this very dependence also provides a means of challenging such definitions. We have seen how generations of writers, scholars and scientists have turned to the animal in order to question, at its very core, the nature of suicide.

For the Romantics, the celebrated case of the defiant and rebellious scorpion was a means of interpreting suicide not as a sinful but a just, even heroic, act. In transferring the scorpion from poetry to experimental laboratory, late nineteenth-century scientists and physicians sought to remove the subject of suicide further from the realm of ethics and morality, and place it under their own jurisdiction. Suicide was not so much a matter for the legal or clerical profession; it was a natural, medical and social problem of genuine and increasing significance. In challenging boundaries between humanity and the natural world in the context of mind and emotion, the suicidal animal was also attractive as it extended a scientific, and specifically Darwinian, understanding into areas considered most sacred and profane, into the heart of religious jurisdiction. There remained a certain Romantic element in play here: humans were not so much debased as the animal promoted — its urge to self-destruction reflecting genuine emotions of grief, anger and love, and therefore reason, intelligence and mind. This is reflected in the sympathetic uses made of animal suicide by those concerned with the maltreatment of animals.

Even when such anthropomorphism was challenged by the development of a more rigorous comparative psychology based on systematic observation and the experimental method, this did not mean the end of the suicidal animal. Its influence endured with its redefinition. The lemming became the archetypal suicidal animal in the twentieth century precisely because of its lack of intelligence, foresight and consciousness. Lemmings were used to describe the senseless devastation of global warfare and to warn of the violence of totalitarian political systems. As the century progressed, the collective impulse became more prominent, and the lemming became less a self-destroyer and more of an automaton. The lemming became the totemic animal in an age of cultural pessimism, a symbol of an unconscious and mindless urge towards mass self-destruction, and references to its suicide are legion.

Simultaneously, interest in explaining lemming behaviour led once again to the animal laboratory. The boundaries between human beings and the natural world were challenged, but now in a more sophisticated way and with a zoomorphic sensibility. Suicide was understood as the outcome of a large variety of other behaviours and disorders, and through the study of a variety of animals — lemmings, rats, dogs and monkeys — such behaviours could now be created, analysed, understood, even ameliorated, in a range of experimental situations. The sciences of ecology, physiology, psychology and psychiatry were imbued with, and connected by, the language of stress. The systems of physiology and behaviour, body and population, were united, making their functions predictable and intervention possible. The understanding gained through animal modelling had significant implications for the study of human beings. For scientists in these disciplines, suicide was a singularly human act only because our definition made it so. By identifying self-destructive behaviours in non-human animals, they were again able to challenge what they interpreted as culturally determined and value-laden definitions of suicide as a unitary, intentional and wilful act. Nature and its systems demanded our understanding, no matter how often it served as a state from which we seemed determined to emancipate ourselves.

## Footnotes

^1^ Emile Durkheim, *Suicide: A Study in Sociology*, trans. John A. Spaulding and George Simpson (London, 2002), 262. Although Morselli served as an important influence on Durkheim, historians have tended to neglect his work. One notable exception is Ty Geltmaker, *Tired of Living: Suicide in Italy from National Unification to World War I, 1860–1915* (New York, 2002).

^2^ Durkheim, *Suicide*, 283.

^3^ Olive Anderson, *Suicide in Victorian and Edwardian England* (Oxford, 1987).

^4^ Stephen Turner, ‘Durkheim among the Statisticians’, *Journal of the History of the Behavioral Sciences*, xxxii (1996), 357.

^5^ Daryl Lee, ‘Accounting for Suicide: Morselli, Moral Statistics and the Modernity of Suicide’, *Intellectual History Review*, xix (2009), 337.

^6^ Durkheim, *Suicide*, xlii. Durkheim defined ‘negative’ acts as the intentional refusal to eat or drink, which he believed were ‘as suicidal as self-destruction by dagger or firearm’.

^7^ *Ibid*., xlii.

^8^ *Ibid*., xliii.

^9^ *Ibid*.

^10^ Albert Camus, *The Myth of Sisyphus*, trans. Justin O’Brien (London, 2005), 40 (emphasis in original). Indeed, though there is not space to explore it here, reflection upon animals underpinned the long-standing Christian strictures against suicide. See Anton J. L. Van Hooff, *From Autothanasia to Suicide: Self-Killing in Classical Antiquity* (London, 1990), xiii.

^11^ Jacques Derrida, *Of Grammatology*, trans. Gayatri Chakravorty Spivak (Baltimore, 1997), 144–54.

^12^ Erica Fudge, *Brutal Reasoning: Animals, Rationality, and Humanity in Early Modern England* (Ithaca, 2006), 6. See also Erica Fudge, ‘A Left-Handed Blow: Writing the History of Animals’, in Nigel Rothfels (ed.), *Representing Animals* (Indianapolis, 2000).

^13^ Esther Cohen, ‘Law, Folklore and Animal Lore’, *Past and Present*, no.110 (Feb. 1986); Fudge, *Brutal Reasoning*; Keith Thomas, *Man and the Natural World: Changing Attitudes in England, 1500–1800* (London, 1987).

^14^ Barbara T. Gates, *Victorian Suicide: Mad Crimes and Sad Histories* (Princeton, 1988).

^15^ Thomas, *Man and the Natural World*, 67.

^16^ Lord Byron, *The Giaour: A Fragment of a Turkish Tale*, 9th edn (London, 1814).

^17^ Kathleen Kete, *The Beast and the Boudoir: Petkeeping in Nineteenth-Century Paris* (Berkeley, 1994); Thomas, *Man and the Natural World*.

^18^ Anon, ‘A Provincial Paper Mentions that a Fine Dog of the Newfoundland Breed Committed Suicide, the Other Day, by Drowning’, *The Satirist; or, the Censor of the Times*, 2 February 1845, 35.

^19^ Edward Jesse, *Anecdotes of Dogs* (London, 1858), 145.

^20^ Anon, ‘Remarkable Case of Suicide in a Dog’, *The Animal World*, iii (1871), 91.

^21^ *Ibid*. For more on the RSPCA and nineteenth-century anti-cruelty campaigns, see Harriet Ritvo, *The Animal Estate: The English and Other Creatures in the Victorian Age* (Cambridge, Mass., 1987).

^22^ Anon, ‘Stag Hunting’, *The Animal World*, 6 (1875), 2.

^23^ Charles Darwin, *The Descent of Man, and Selection in Relation to Sex* (London, 2004), 151.

^24^ Francis Power Cobbe to Charles Darwin, 25 December 1872: Archives and Manuscripts, University of Cambridge, DAR 161:189.

^25^ W. Lauder Lindsay, ‘Physiology of Mind in the Lower Animals’, *Journal of Mental Science*, xvii (1871), 79 (emphasis in original).

^26^ *Ibid*., 76 (emphasis in original).

^27^ Anon., ‘Obituary: William Lauder Lindsay’, *British Journal of Psychiatry*, xxvi (1881), 643. On John Conolly’s contributions to psychiatric and asylum reform, see Andrew Scull, ‘A Victorian Alienist: John Conolly, FRCP, DCL (1794–1866)’, in W. F. Bynum, Roy Porter and Michael Shepherd (eds.), *The Anatomy of Madness: Essays in the History of Psychiatry*, i, *People and Ideas* (London, 1985). Despite his public association with the reforming ideals of Conolly, Lindsay nevertheless believed that mechanical restraint was sometimes necessary for the protection of both patient and staff. See W. Lauder Lindsay, ‘The Protection Bed and Its Uses’, *Edinburgh Medical Journal*, xxiii (1878).

^28^ W. Lauder Lindsay, ‘Madness in Animals’, *Journal of Mental Science*, xxiii (1871), 195 (emphasis in original).

^29^ See Barbara T. Gates, ‘Suicide and the Victorian Physicians’, *Journal of the History of the Behavioral Sciences*, xvi (1980).

^30^ Lindsay, ‘Madness in Animals’, 185 (emphasis in original). See also W. Lauder Lindsay, *Physiology and Pathology of Mind in the Lower Animals* (Edinburgh, 1871), 12–14.

^31^ W. Lauder Lindsay, *Mind in the Lower Animals in Health and Disease*, ii, *Mind in Disease* (London, 1879), 130 (emphasis in original). See also Lindsay, *Physiology and Pathology of Mind in the Lower Animals*, 2.

^32^ Lindsay, *Physiology and Pathology of Mind in the Lower Animals*, 12.

^33^ On Maudsley’s influence, see Gates, ‘Suicide and the Victorian Physicians’, 169–70.

^34^ Henry Maudsley, ‘The Genesis of Mind’, *Journal of Mental Science*, vii (1862); Henry Maudsley, ‘The Genesis of Mind [continuation]’, *Journal of Mental Science*, viii (1862).

^35^ Maudsley, ‘Genesis of Mind [continuation]’, 73, 84.

^36^ Henry Maudsley, ‘Alleged Suicide of a Dog’, *Mind*, iv (1879), 413.

^37^ *Ibid*., 411.

^38^ Anon, ‘*Mind in the Lower Animals*, by W. Lauder Lindsay, M.D.’, *Journal of Mental Science*, xxvi (1880), 280. This anonymous review is highly likely to have been written by Maudsley, who reviewed many books for the *Journal of Mental Science*, since it repeats many of the arguments made in his articles on ‘The Genesis of Mind’ and ‘Alleged Suicide of a Dog’.

^39^ In line with the prevailing belief that suicidal acts necessitated the mental awareness of death, and the ability to foresee and carry out a plan, many writers believed that deliberate suicide was extremely rare among groups with ‘inferior’ mental capabilities such as young children, the mentally ill and ‘primitive peoples’. See, for example, Henry Morselli, *Suicide: an Essay on Comparative Moral Statistics* (London, 1883), 205.

^40^ Anon, ‘*Mind in the Lower Animals*’, 280.

^41^ W. Lauder Lindsay, *Mind in the Lower Animals in Health and Disease*, i, *Mind in Health* (London, 1879), 18.

^42^ *Ibid*., xiii.

^43^ *Ibid*., 26. On the growth of the experimental ethos in nineteenth-century biology, see Gerald L. Geison, *Michael Foster and the Cambridge School of Physiology: the Scientific Enterprise in Late Victorian Society* (Princeton, 1978).

^44^ Lindsay, *Mind in the Lower Animals in Health and Disease*, i, 25–6.

^45^ *Ibid*., 26.

^46^ *Ibid*., 32.

^47^ *Ibid*., 29–36.

^48^ *Ibid*., 30 (emphasis in original).

^49^ *Ibid*., 34–5.

^50^ *Ibid*., 57.

^51^ Alfred G. Bourne, ‘The Reputed Suicide of Scorpions’, *Proceedings of the Royal Society of London*, xlii (1887), 18.

^52^ G. Bidie, ‘Suicide of a Scorpion’, *Nature*, ix (1874), 29 (emphasis in original).

^53^ *Ibid*.

^54^ Allen Thomson, ‘Suicide of the Scorpion’, *Nature*, xx (1879), 577.

^55^ Gates, *Victorian Suicide*; Anderson, *Suicide in Victorian and Edwardian England*.

^56^ William Wynn Westcott, *Suicide: Its History, Literature, Jurisprudence, Causation and Prevention* (London, 1885), 176, A2.

^57^ *Ibid*., 176.

^58^ Robert Boakes, *From Darwin to Behaviourism: Psychology and the Minds of Animals* (Cambridge, 1984), 25–32.

^59^ George J. Romanes, *Animal Intelligence* (London, 1882), 225.

^60^ For more detail, see Gregory Radick, *The Simian Tongue: the Long Debate about Animal Language* (Chicago, 2007), 7; Boakes, *From Darwin to Behaviourism*, 25–32.

^61^ Morgan believed that the human mind was fundamentally different from the animal mind on account of its capacity for abstract thought which, he believed, was clearly evidenced by language. See Radick, *Simian Tongue*, 75.

^62^ C. Lloyd Morgan, ‘Suicide of Scorpions’, *Nature*, xxvii (1883), 530.

^63^ *Ibid*., 313.

^64^ *Ibid*.

^65^ Radick, *Simian Tongue*, 51.

^66^ *Ibid*.

^67^ Maudsley, ‘Genesis of Mind’, 483.

^68^ *Ibid*., 97.

^69^ *Ibid*., 88.

^70^ Radick, *Simian Tongue*, 207–11.

^71^ See Daniel P. Todes, *Pavlov’s Physiological Factory: Experiment, Interpretation, Laboratory Enterprise* (Baltimore, 2002); Eileen Crist, *Images of Animals: Anthropomorphism and Animal Mind* (Philadelphia, 1999).

^72^ Durkheim, *Suicide*, 39.

^73^ *Ibid*., 246.

^74^ Peter Hamilton, ‘Commentary’, in Peter Hamilton (ed.), *Emile Durkheim: Critical Assessments*, iii (London, 1989), 4.

^75^ Morselli, *Suicide*, 1.

^76^ *Ibid*., 3.

^77^ *Ibid*., 8.

^78^ *Ibid*., 118.

^79^ *Ibid*., 364.

^80^ S. A. K. Strahan, *Suicide and Insanity: a Physiological and Sociological Study* (London, 1893), 26.

^81^ *Ibid*., 69.

^82^ *Ibid*., 68. This association between the savage and the lower animal was common in discussions of suicide, but did not go unchallenged, particularly by anthropologists and ethnologists. See S. R. Steinmetz, ‘Suicide among Primitive People’, *American Anthropologist*, vii (1894).

^83^ Strahan, *Suicide and Insanity*, 102.

^84^ *Ibid*., 69.

^85^ John C. Weaver, *A Sadly Troubled History: the Meanings of Suicide in the Modern Age* (Montreal, 2009), 32–4. We do not mean to suggest that there were not very significant differences between these approaches. Strahan, for example, believed that the true suicide was mentally ill, in direct contrast to Durkheim.

^86^ See, for example, Louis I. Dublin and Bessie Bunzel, *To Be or Not to Be: a Study of Suicide* (New York, 1933).

^87^ On Pearl’s statistical and biological determinism and his vision of an interdisciplinary population biology, see Edmund Ramsden, ‘Carving up Population Science: Eugenics, Demography and the Controversy over the “Biological Law” of Population Growth’, *Social Studies of Science*, xxxii (2002).

^88^ Lawrence G. Green, ‘Science Puzzling Over a New Epidemic of Race Suicide by Whales’, *Albuquerque Journal*, 6 Dec. 1936.

^89^ Particularly influential was the work of the Norwegian natural historian Robert Collett, which was translated into English. See Robert Collett, ‘On *Myodes lemmus* in Norway’, *The Journal of The Linnean Society, Zoology*, xiii (1877); R. Collett, *Myodes lemmus, its Habits and Migrations in Norway* (Christiana, 1895).

^90^ Anon., ‘The Lemming, or Migratory Rat’, *The Treasury of Literature and The Ladies’ Treasury*, 1 Nov. 1871.

^91^ In the 1871 essay, the lemming is described as ‘pre-eminently a devastator … An enemy so numerous and destructive would quickly render the countries they devastated barren’. See *Ibid*.

^92^ N. C. Stenseth and R. A. Ims, ‘The History of Lemming Research: from the Nordic Sagas to *The Biology of Lemmings*’, in N. C. Stenseth and R. A. Ims (eds.) *The Biology of Lemmings* (London, 1993), 21.

^93^ Brooke was reiterating an eye-witness account from a Mr Knudtzon of Drontheim. A. de Capell Brooke, ‘Travels through Sweden, Norway, and Finmark to the North Cape, in the Summer of 1820’, *Quarterly Review*, xxx (1823), 130.

^94^ Lindsay, *Mind in the Lower Animals*, i, 151. See also W. Lauder Lindsay, ‘Mental Epidemics among Lower Animals’, *Journal of Mental Science*, xvii (1872).

^95^ Pearl spoke of the history of humanity presenting ‘objectively a rather remarkable parallelism to the successive stages (up to the final one) in the history of a lemming population’, which is, of course, ultimately destructive to large sections of the population: Raymond Pearl, ‘On Biological Principles Affecting Populations: Human and Other’, *The American Naturalist*, lxxi (1937), 64.

^96^ Peter Crowcroft, *Elton’s Ecologists: A History of the Bureau of Animal Population* (Chicago, 1991), 3.

^97^ C. S. Elton, ‘Periodic Fluctuations in Numbers of Animals: their Causes and Effects’, *British Journal of Experimental Biology*, ii (1924).

^98^ Charles Elton, *Voles, Mice, and Lemmings: Problems in Population Dynamics* (Oxford, 1942), 214. Elton noted how lemming migration had ‘been given a tinge of epic romance’ by the poets laureate Robert Bridges and John Masefield. Bridges, in his poem *The Testament of Beauty*, compared their migration to the second crusade. He also described the lemming migration as ‘analogous to infanticide among human beings as a method of preventing overpopulation’ (Elton, ‘Periodic Fluctuations in Numbers of Animals’, 126). For a historical analysis of the attempts to understand the lemming cycle, see Stephen Bocking, ‘Science and Spaces in the Northern Environment’, *Environmental History*, xii (2007); Dennis Chitty, *Do Lemmings Commit Suicide? Beautiful Hypotheses and Ugly Facts* (Oxford, 1996).

^99^ Northrop Frye, ‘*The Decline of the West* by Oswald Spengler’, *Daedalus*, ciii (1974), 9.

^100^ Quoted in John Brooke, ‘Namier and Namierism’, *History and Theory*, iii (1964). Namier was drawing on Freud’s group psychology and shared his concern that mass behaviour was a reflection of mankind’s more primitive urges. Sigmund Freud, *Group Psychology and the Analysis of the Ego*, trans. James Strachey (London, 1959). See Daniel Pick, ‘Freud’s *Group Psychology* and the History of the Crowd’, *History Workshop Journal*, xl (1995).

^101^ Erich Fromm, *The Anatomy of Human Destructiveness* (Harmondsworth, 1977), 462. The case of the lemming seemed relevant to survival after a nuclear attack, reflecting its application to both aggressor and victim. For Fromm the effects of nuclear war would be ‘mass psychosis’ and the re-emergence of ‘a new form of primitive barbarism, to the resurgence of the most archaic elements, which are still potentialities in every man and of which we have had ample evidence in the terror systems of Hitler and Stalin’. Erich Fromm, ‘The Case for Unilateral Disarmament’, *Daedalus*, lxxxix (1960), 1018.

^102^ Erich Fromm, ‘The Present Human Condition’, *The American Scholar*, xxv (1955–56), 33.

^103^ See, for example, Karl Menninger, ‘The Suicidal Intention of Nuclear Armament’, *Bulletin of the Menninger Clinic*, xlvii (1983); Nicholas Humphrey, *Four Minutes to Midnight: the Bronowski Memorial Lecture 1981* (London, 1982); Hudson Hoagland, ‘Biological Aspects of Aggression and Violence’, *Zygon*, iv (1969); Lawrence S. Kubie, ‘The Eagle and the Ostrich’, *Archives of General Psychiatry*, v (1961).

^104^ Bruno Bettelheim, ‘Foreword’, in Miklos Nyiszli, *Auschwitz: a Doctor’s Eyewitness Account*, trans. Tibère Kremer and Richard Seaver (New York, 1960), vi–vii.

^105^ Bruno Bettelheim, ‘Individual and Mass Behavior in Extreme Situations’, *Journal of Abnormal and Social Psychology*, xxxviii (1943).

^106^ Karl Menninger, ‘Psychoanalytic Aspects of Suicide’, *International Journal of Psychoanalysis*, xiv (1933); Karl Menninger, *Man against Himself* (London, 1938).

^107^ Gregory Zilboorg, ‘Suicide among Civilized and Primitive Races’, *American Journal of Psychiatry*, xcii (1936).

^108^ *Ibid*.

^109^ On this point, see George Simpson, ‘Methodological Problems in Determining the Aetiology of Suicide’, *American Sociological Review*, xvi (1950); Lawrence S. Kubie, ‘Multiple Determinants of Suicide’, in Edwin S. Shneidman (ed.), *Essays in Self-Destruction* (New York, 1967).

^110^ Sigmund Freud, *Beyond the Pleasure Principle*, trans. James Strachey (New York, 1959), 308.

^111^ *Ibid*., 311.

^112^ This ranged from the amputation of limbs and sexual organs to hair-pulling and nail-biting. Karl Menninger, ‘A Psychoanalytic Study of the Significance of Self-Mutilations’, *Psychoanalytic Quarterly*, iv (1935).

^113^ Menninger, *Man against Himself*, 7; Menninger, ‘A Psychoanalytic Study of the Significance of Self-Mutilations’, 456; Karl Menninger, ‘The Aacdemic Lecture: Hope’, *American Journal of Psychiatry*, cxvi (1959).

^114^ ‘Notes’ in *Psychoanalytic Quarterly*, vi (1937), 273; vii (1938), 167–8; ix (1940), 160. The committee was originally supported by Marshall Field, a banker, and comprised seven psychiatrists and two social workers. It was disbanded with the entry of the United States into World War II.

^115^ The death instinct proved Freud’s most controversial hypothesis: ‘the most bizarre monster of all Freud’s gallery of monsters’ according to William McDougall, quoted in John Flugel, ‘The Death Instinct, Homeostasis and Allied Concepts’, *International Journal of Psychoanalysis, Supplement*, xxxiv (1953), 43. For Freud’s critics, the fact that the instinct seemed based in biology, shared by ‘all organic life’, the lack of evidence of a self-destructive impulse among animals was, they argued, damning. For Sylvia Payne, the death instinct was ‘difficult for scientists to accept’: ‘If we turn to biology for evidence, the question arises as to whether we can observe a death instinct in animals. As a rule animals are aggressive in order to preserve life’. Pearl King and Riccardo Steiner (eds.), *The Freud–Klein Controversies, 1941–45* (London, 1991), 746. Freud did draw on some biological evidence, particularly the ‘unexpected analogy’ with Auguste Weismann’s theory that the body, or *soma*, was programmed to die to ensure the immortality of the germ-plasm. See Freud, *Beyond the Pleasure Principle*, 81. For criticism of his reliance on Weisman, see C. P. Blacker, ‘Life and Death Instincts’, *British Journal of Medical Psychology*, ix (1929). In his criticism, Blacker drew on Morgan’s study of the scorpion, and argued that the idea of lemming suicide would run counter to Darwinian evolution. Blacker did, however, propose that animals could, like man, die from ‘inanition’ when placed in an unfavourable environment, and that suicide itself resulted from the misdirection of useful biological instincts, such as aggression, but not from a specific ‘death impulse’.

^116^ Gregory Zilboorg, ‘Considerations on Suicide, with Particular Reference to that of the Young’, *American Journal of Orthopsychiatry*, vii (1937), 22.

^117^ *Ibid*., 23.

^118^ *Ibid*., 23–4. See also Gregory Zilboorg, ‘Differential Diagnostic Types of Suicide’, *Archives of Neurology and Psychiatry*, xxxv (1936).

^119^ Zilboorg, ‘Considerations on Suicide’, 28–9.

^120^ *Ibid*., 15.

^121^ *Ibid*., 18.

^122^ See, for example, Gregory Zilboorg, ‘Sociology and the Psychoanalytic Method’, *American Journal of Sociology*, xlv (1939); Maurice Halbwachs, *Les Causes du suicide* (Paris, 1930).

^123^ Gregory Zilboorg, ‘The Sense of Reality’, *Psychoanalytic Quarterly*, x (1941).

^124^ W. Duppa Crotch, ‘On the Migration and Habits of the Norwegian Lemming’, *The Journal of The Linnean Society, Zoology*, xiii (1876), 34.

^125^ For an analysis of the Rodent Ecology Project, see Christine Keiner, ‘Wartime Rat Control, Rodent Ecology, and the Rise and Fall of Chemical Rodenticides’, *Endeavour*, xxix (2005).

^126^ John B. Calhoun, *The Ecology and Sociology of the Norway Rat* (Bethesda, Md, 1963).

^127^ Calhoun had received direct encouragement from Pearl in this regard, who wrote to him in 1940, when Calhoun was a PhD student in zoology at Northwestern. See John B. Calhoun, ‘Looking Backward from “The Beautiful Ones” ’, in W. R. Klemm (ed.), *Discovery Processes in Modern Biology* (Melbourne, Fla., 1977).

^128^ The most famous of Calhoun’s publications was based on this later research with rats at the NIMH: John B. Calhoun, ‘Population Density and Social Pathology’, *Scientific American*, cccvi (1962). For a history of Calhoun’s research and influence, see Edmund Ramsden and Jon Adams, ‘Escaping the Laboratory: the Rodent Experiments of John B. Calhoun and their Cultural Influence’, *Journal of Social History*, xlii (2009).

^129^ Halsey M. Marsden, ‘Crowding and Population Density’, in Joachim F. Wohlwill and Daniel Carson (eds.), *Environment and the Social Sciences: Perspectives and Applications* (Washington DC, 1972), 9.

^130^ J. B. Calhoun, ‘The Theoretical Framework from which I Approach Problems of Group Behavior’, Miscellaneous documents — set I, volume II — 1953–55 (folder 1 of 2), 1955: John B. Calhoun Papers, National Library of Medicine, Bethesda, Maryland.

^131^ John J. Christian, ‘The Adreno-Pituitary System and Population Cycles in Mammals’, *Journal of Mammology*, xxi (1950).

^132^ Dennis Chitty, ‘Population Processes in the Vole and their Relevance to General Theory’, *Canadian Journal of Zoology*, xxxviii (1960). On the British programme of rodent control, see Dennis Chitty and H. N. Southern (eds.), *Control of Rats and Mice*, 3 vols. (Oxford, 1954).

^133^ Thomas Hornsby Ferril, ‘Western Half-Acre’, *Harper’s Magazine*, cxci (1945), 534.

^134^ For René Dubos, the population crashes experienced by the lemming revealed nature to be short-sighted, clumsy and defective: ‘Judging from the point of view of lemmings, muskrats, and rabbits — let alone human beings — only the most starry-eyed Panglossian optimist could claim that nature knows best how to achieve population control’. See René J. Dubos, ‘Humanizing the Earth’, *Science*, clxxix (1973), 769. For a critique of the balance of nature concept, with which Calhoun would have had much sympathy, see P. R. Ehrlich and L. C. Birch, ‘The “Balance of Nature” and “Population Control” ’, *The American Naturalist*, ci (1967).

^135^ John B. Calhoun, ‘The Lemmings’ Periodic Journeys are not Unique’, *Smithsonian Magazine*, i (1971).

^136^ Marsden, ‘Crowding and Population Density’, 7.

^137^ Calhoun’s 1971 paper on the lemmings was presented as evidence of the dangers of population growth and the need for government intervention by Senator Robert Packwood, *Congressional Record*, 1 April 1971, 92nd congress, 1st session.

^138^ For a detailed analysis of the growth of the field of environmental stress, and the role of crowding within it, see Edmund Ramsden, ‘From Rodent Utopia to Urban Hell: Population, Pathology, and the Crowded Rats of NIMH’, *Isis*, cii (2011).

^139^ D. H. Stott, ‘Abnormal Mothering as a Cause of Mental Subnormality — II. Case Study and Conclusions’, *Journal of Child Psychology and Psychiatry*, iii (1962).

^140^ D. H. Stott, ‘Cultural and Natural Checks on Population-Growth’, in M. F. Ashley Montagu, (ed.), *Culture and the Evolution of Man* (New York, 1962), 365–6.

^141^ Denis Stott, *Delinquency: the Problem and its Prevention* (London, 1982), 201.

^142^ *Ibid*.

^143^ Stott, ‘Abnormal Mothering as a Cause of Mental Subnormality’, 145.

^144^ Paul B. Paulus and Garvin McCain, ‘Crowding in Jails’, *Basic and Applied Social Psychology*, iv (1983), 89. See also Paul B. Paulus, *Prison Crowding: a Psychological Perspective* (New York, 1988).

^145^ Ivor H. Mills, ‘The Human Lemmings: Escape From Realities of Life’, *The Times*, 30 May 1970.

^146^ Zilboorg, ‘Considerations on Suicide’, 24.

^147^ Jules H. Masserman, *Behavior and Neurosis: an Experimental Psychoanalytic Approach to Psychobiologic Principles* (Chicago, 1943), 6–7.

^148^ Jules H. Masserman, ‘Comparative and Clinical Approaches to Biodynamic Therapy’, in Jules H. Masserman, (ed.), *Animal and Human: Scientific Proceedings of the American Academy of Psychoanalysis* (New York, 1968), 253.

^149^ Jules H. Masserman, *Principles of Dynamic Psychiatry* (Philadelphia, 1961), 195.

^150^ Israel Hanin and Earl Usdin, ‘Foreword’, in I. Hanin and E. Usdin (eds.), *Animal Models in Psychiatry and Neurology* (Rockville, 1977), xiii.

^151^ William T. McKinney Jr, ‘Behavioral Models of Depression in Monkeys’, in Hanin and Usdin, (eds.), *Animal Models in Psychiatry and Neurology*, 117.

^152^ *Ibid*.

^153^ Laurens D. Young, Stephen S. Suomi, Harry Harlow and William T. McKinney Jr, ‘Early Stress and Later Response to Separation in Rhesus Monkeys’, *American Journal of Psychiatry*, cxxx (1973).

^154^ Harry F. Harlow, Robert O. Dodsworth and Margaret K. Harlow, ‘Total Social Isolation in Monkeys’, *Proceedings of the National Academy of Sciences of the United States of America*, liv (1965), 92.

^155^ Harry F. Harlow, Philip E. Plubell and Craig M. Baysinger, ‘Induction of Psychological Death in Rhesus Monkeys’, *Journal of Autism and Childhood Schizophrenia*, iii (1973), 299.

^156^ Harlow, Dodsworth and Harlow, ‘Total Social Isolation in Monkeys’, 95, 90.

^157^ Ivor H. Jones, ‘Self-injury: toward a Biological Basis’, *Perspectives in Biology and Medicine*, xxvi (1982), 138.

^158^ Jones, ‘Self-injury’, 140.

^159^ Thomas Joiner, *Why People Die by Suicide* (Cambridge, Mass., 2005), 115.

^160^ Bruno Bettelheim, *The Empty Fortress: Infantile Autism and the Birth of the Self* (New York, 1967).

^161^ Karl A. Menninger, ‘Psychological Aspects of the Organism Under Stress — Part II, Regulatory Devices of the Ego Under Major Stress’, *Journal of the American Psychoanalytic Association*, ii (1954), 297. It is important to note that not all psychoanalysts viewed animal research with such unanimity. Many saw it as a direct challenge to their authority, particularly with the rise of behaviour therapy as an alternative to psychotherapy in the 1960s. The president of the American Psychoanalytic Association, Leo Rangell, spoke of a growing ‘disenchantment’ with psychoanalysis — ‘Anything else is seized upon as furnishing an alternative “explanation”, whether Lorenz’s imprinting, Harlow’s experimentally treated primates … or the behavior of dolphins or flies’. See Leo Rangell, ‘Psychoanalysis — A Current Look’, *Bulletin of the American Psychoanalytic Association*, xxiii (1967). Evidence of the primary importance of touch between mother and infant in Harlow’s studies was an important challenge to Freudian ideas which interpreted it as a secondary phenomenon resulting from need satisfaction.

^162^ John Bowlby, ‘Disruption of Affectional Bonds and its Effects on Behavior’, *Journal of Contemporary Psychotherapy*, ii (1970), 75. See also John Bowlby, ‘Processes of Mourning’, *International Journal of Psychoanalysis*, xlii (1961), in which he suggested that the processes of grief and despair were shared by dogs, geese and monkeys, concluding that ‘the presence of hostility directed towards the self’ was not specific to man. Bowlby was critical of notions such as the ‘death instinct’, as it was ‘rooted in a non-evolutionary paradigm that bears no relation to modern biology’ — John Bowlby, *Attachment and Loss*, ii, *Separation: Anxiety and Anger* (London, 1975), 205. In turn, Harlow and his associates presented their studies of ‘separation stress’ as providing much-needed animal models for human psychopathology, particularly depression, attempted suicide, suicidal impulses and ‘despair’, as studied by Bowlby. (See Young, Suomi, Harlow and McKinney, ‘Early Stress and Later Response to Separation in Rhesus Monkeys’, 401.)

^163^ Jones, ‘Self-injury’, 141.

^164^ Jones, ‘Self-injury’, 146. For a similar comparison between self-mutilation among experimental monkeys and among overcrowded prisoners experiencing alienation within ‘the lonely crowd’, see Armando R. Favazza, *Bodies under Siege: Self-Mutilation and Body Modification in Culture and Psychiatry* (Baltimore, 1992), 77. Bernice Einsidler and L. D. Hankoff similarly argue that death from the stress of the crowd, hastened by self-injury, reveals the term suicide to be of ‘dubious scientific usefulness, incorporating as it does the notion of choice or intent’. Suicidal behaviour, they argue, does not require ‘a knowledge or concept of self’. ‘Self-Injury in Animals’, in Bernice Einsidler and L. D. Hankoff (eds.), *Suicide: Theory and Clinical Aspects* (Littleton, 1979), 138, 139.

^165^ Jones, ‘Self Injury’, 148.

^166^ I. H. Jones and B. M. Barraclough, ‘Auto-Mutilation in Animals and its Relevance to Self-Injury in Man’, *Acta Psychiatrica Scandinavica*, lviii (1978) 40–7.

^167^ On this point, see David Lester, ‘The Categorization of Suicide’, *The Behavioral and Brain Sciences*, iii (1980), 281.

^168^ For reviews of the use of animal models of suicidal behaviour, see Jacqueline N. Crawley, Mary E. Sutton and David Pickar, ‘Animal Models of Self-destructive Behavior and Suicide’, *Psychiatric Clinics of North America*, viii (1985); David M. Stoff and J. John Mann, ‘Suicide Research: Overview and Introduction’, *Annals of the New York Academy of Sciences*, dcccxxxvi (1997); Robert D. Goldney, ‘Ethology and Suicidal Behaviour’, in Keith Hawton and Kees van Heeringen (eds.), *The International Handbook of Suicide and Attempted Suicide* (Chichester, 2000); Antonio Preti, ‘Animal Model and Neurobiology of Suicide’, *Progress in Neuro-Psychopharmacology and Biological Psychiatry*, xxxv (2011); Oz Malkesman *et al.*, ‘Animal Models of Suicide-Trait-Related Behaviors’, *Trends in Pharmacological Sciences*, xxx (2009).

^169^ Edward O. Wilson, *Sociobiology: the New Synthesis* (Cambridge, Mass., 1975). Wilson argued that one would see countless instances of self-destruction in the animal kingdom — evolutionary adaptations that benefit a population. For his critics, such as the population geneticist, Richard Lewontin, ‘it is pure metaphor’, he argued, ‘to describe numbers of chromosomes, patterns of fertility, migrations, and religious institutions as “adaptations”. It is this kind of error that led to the now discredited descriptions of lemming “migrations” and “mass suicides” as adaptive responses to crowding’. R. C. Lewontin, ‘On Constraints and Adaptation’, *The Behavioral and Brain Sciences*, ii (1981), 245. For Lewontin the suicidal lemming assumed pride of place in his dismissal of the excessive search for adaptive explanation as a ‘test of the ingenuity of theorists and of the tolerance of intellectuals for tortured and absurd stories’. Richard Levins and Richard Lewontin, *The Dialectical Biologist* (Boston, 1985), 73. For a more recent sociobiological treatment that suggests suicide has an adaptive value, removing (through stress) those who are burdens and childless, see Denys deCatanzaro, ‘Human Suicide: a Biological Perspective’, *The Behavioral and Brain Sciences*, iii (1980). While deCatanzaro assesses, but rejects, a number of cases of self-destruction among animals as suicide, some of his commentators do not: ‘It appears very likely that suicide may occur in animals in the absence of a knowledge or concept of self’: F. V. Wenz, ‘Heredity, Environment, and Culture in Suicide’, *The Behavioral and Brain Sciences*, iii (1980).

^170^ The case of the lemming’s behaviour continues to be debated by scientists, but few see it as the consequence of a suicidal urge. Central to the debate is whether population dynamics are determined by self-regulatory processes such as stress or by the lemmings’ migration in search of dwindling food supplies. See, for example, Chitty, *Do Lemmings Commit Suicide?*

^171^ Edwin Shneidman, *Suicide as Psychache: a Clinical Approach to Self-Destructive Behavior* (Northvale, 1993), 5.

^172^ For example, in an introductory psychology textbook, Philip Bell and Phillip Staines argue: ‘The tendency to anthropomorphism is very great when apparently inexplicable biological phenomena appear to be like those of human society: hence the widely held interpretation of the mass migration of lemmings which culminates in their death as an act of “suicide” — an interpretation which a moment’s reflection will show to be very difficult to sustain. For example, does each lemming (a small rodent) consciously “decide” that its life is not worth living?’ Philip Brian Bell and Phillip James Staines, *Reasoning and Argument in Psychology* (London, 1981), 102.

^173^ Jean Baechler, *Suicides*, trans. Barry Cooper (Oxford, 1979), 42.

^174^ Henri Bergson, *The Two Sources of Morality and Religion*, trans. R. Ashley Audra, Cloudesley Brereton and W. Horsfall Carter (London, 1935), 108.

